# Critical investments in bioregenerative life support systems for bioastronautics and sustainable lunar exploration

**DOI:** 10.1038/s41526-025-00518-4

**Published:** 2025-08-16

**Authors:** D. Marshall Porterfield, Dana Tulodziecki, Raymond Wheeler, Mai’a K. Davis Cross, Oscar Monje, Lynn J. Rothschild, Richard J. Barker, Hansjorg Schwertz, Steven Collicott, Som Dutta

**Affiliations:** 1 Department of Agricultural and Biological Engineering, Purdue University, West Lafayette, IN USA; 2 Purdue Policy Research Institute, Purdue University, West Lafayette, IN USA; 3 Department of Philosophy, Purdue University, West Lafayette, IN USA; 4 NASA Kennedy Space Center, Cape Canaveral, FL USA; 5 Department of Political Science, Northeastern University, Boston, MA USA; 6 Air Revitalization Laboratory, Aetos Systems, Kennedy Space Center, Cape Canaveral, FL USA; 7 NASA Ames Research Center, Planetary Systems Branch, Moffett Field, CA USA; 8 Molecular Medicine Program and Division of Occupational Medicine, University of Utah, Salt Lake City, UT USA; 9 Employee Health and Research at Billings Clinic, Bozeman, MT USA; 10 Aerospace and Aeronautical Engineering, Purdue University, West Lafayette, IN USA; 11 Department of Mechanical and Aerospace Engineering, Utah State University, Logan, UT USA

**Keywords:** Decision making, Economics, Government, Politics, Funding, Research management, Biological techniques, Biophysics, Biotechnology, Computational biology and bioinformatics, Molecular biology, Plant sciences, Psychology, Systems biology, Ecology, Environmental sciences, Biomarkers, Health care, Medical research, Risk factors, Engineering, Materials science, Nanoscience and technology, Business and industry, Scientific community

## Abstract

NASA and the CNSA have both released plans for lunar human exploration. This paper reviews those plans through the lens of strategic capability development. It examines the history of NASA’s development of bioregenerative space habitation systems and shows how past research and policy decisions, including funding cuts and program discontinuations, have led to critical gaps in current NASA capabilities. These gaps pose a strategic risk to US leadership in human space exploration that must be addressed urgently to sustain international competitiveness. It concludes with recommendations for program investments crucial for the deployment of mature bioregenerative technologies in the coming decade.

## Introduction

**Fig. 1:**
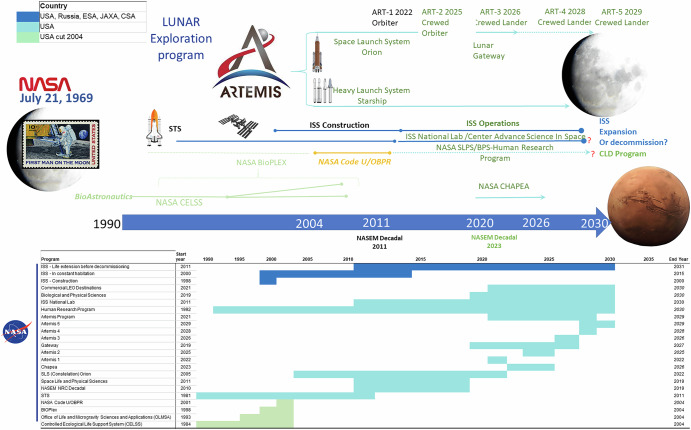
Timeline of past and proposed programmatic development activities for lunar exploration for the National Aeronautics and Space Administration (NASA) of the United States of America with partner nations including Russia, Japan, and the ESA states. These timelines are focused on lunar science and exploration systems for human operations on the lunar surface. This includes both traditional landing missions, such as those conducted by the USA in the Apollo era, and future mission programs based on Bioregenerative Life Support Systems (BLiSS). These timelines reflect dependencies on launch systems, hardware, and the emerging biotech of advanced human habitation.

**Fig. 2:**
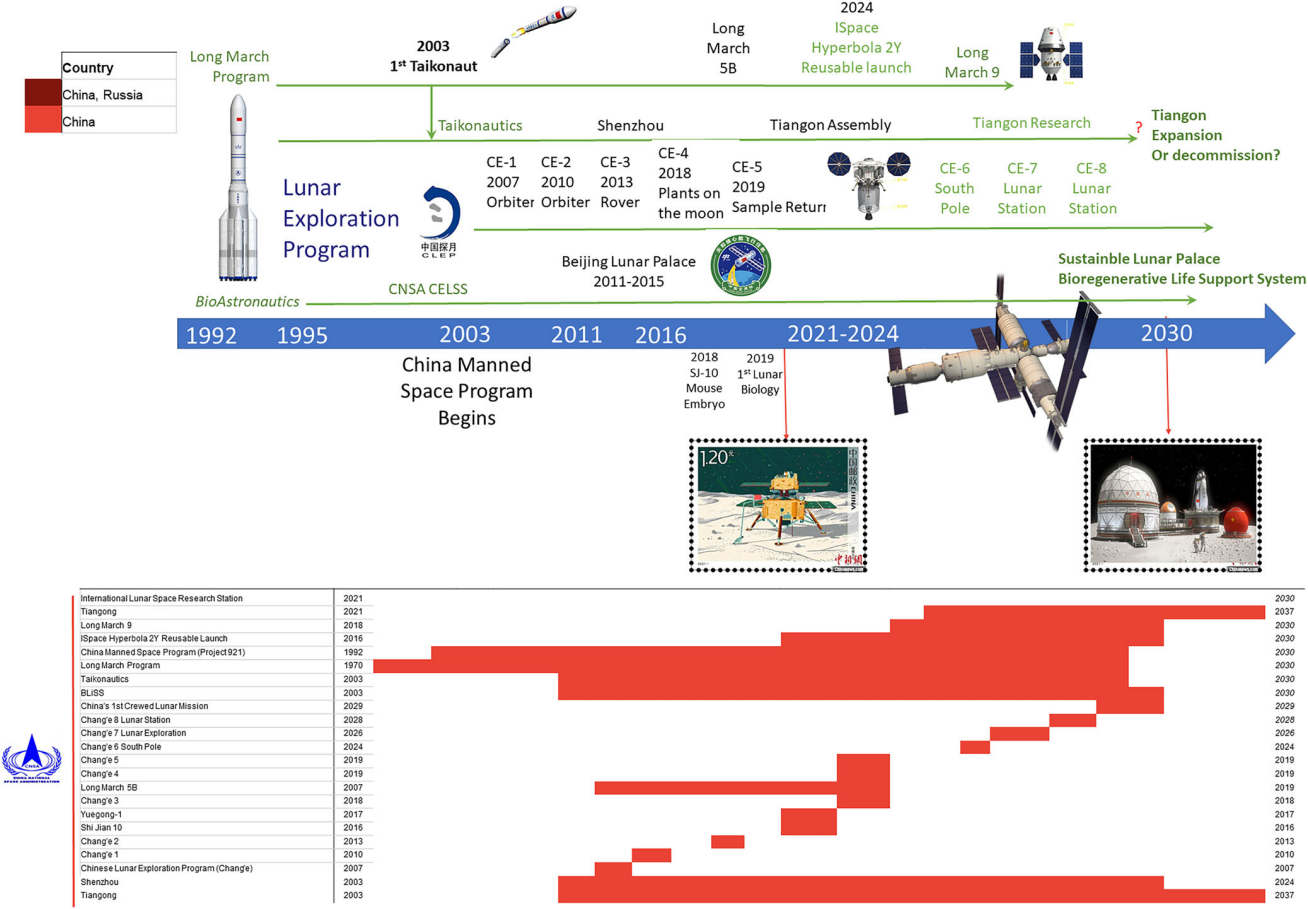
Timeline of past and proposed programmatic development activities for lunar exploration for the China National Space Administration (CNSA). This figure was organized to show timelines focused on lunar exploration systems for human operations on the lunar surface, and future missions and programs based on Bioregenerative Life Support Systems (BLiSS). These timelines reflect dependencies on launch systems, hardware, and the emerging biotech of advanced human habitation. Based on the investments made by the CNSA, the current Chinese program is clearly leading in the development of advanced life support for deep space operations in the coming decade. While the CNSA has only recently entered into the realm of “heavy lift” vehicles, their track record of success in launching and assembling the Tiangong station, as well as new reusable launch systems, demonstrates competency volumetrically.

Logistics costs, technology limits, and human health and safety risks are the trinity that constrain human space exploration operations using current physical/chemical methods for environmental life support to maintain human presence and habitation. Both the National Aeronautics and Space Administration^1^ (NASA) and the China National Space Administration^2–4^ (CNSA) have released public plans for future lunar exploration programs that include long-duration lunar habitation mission capabilities. Current US approaches rely on resupply of food, some water, and other consumable materials required for physical/chemical-based environmental closed loop life support systems (ECLSS). In contrast, earlier approaches were focused on bioregenerative life support by advancing controlled environment agriculture (CEA) for logistically biosustainable exploration, in alignment with historical initiatives like Project Horizon (1959), that emphasized the logistical biosustainability of lunar habitat^5^. Such approaches were the foundation of the NASA Controlled Ecological Life Support Systems (CELSS) program^6,7^, which led to the NASA Bioregenerative Planetary Life Support Systems Test Complex (BIO-PLEX) habitat demonstration program^8^. Discontinued and physically demolished by NASA after the release of the Exploration Systems Architecture Study (ESAS) in 2004^9^, these very same bioregenerative life support programs have been embraced and advanced by China and the CNSA over the last 20 years^10,11^. Many of the canceled NASA technology development programs were incorporated into the CNSA lunar program, most notably in the form of the Beijing Lunar Palace, which was, in addition to domestic innovation, also in part derived from and facilitated by the outputs of the NASA CELSS program^10^. Published NASA BIO-Plex plans supported the CNSA’s efforts to swiftly establish a bioregenerative habitat technology program for an operational human lunar outpost and, subsequently, to demonstrate its viability^4^. The European Space Agency’s (ESA’s) moderate but productive Micro-Ecological Life Support System Alternative (MELiSSA) program is also focused on bioregenerative life support systems (BLiSS, sometimes BLSS) component technology^12^, but it never approached closed-systems human testing. Besides the Chinese efforts, there are currently no other official programs pursuing a fully integrated, closed-loop bioregenerative architecture for establishing lunar or Martian habitats, or even for sustaining long-term human presence in space. By now, the CNSA has therefore taken the lead in this arena, successfully demonstrating closed-system operations for atmosphere, water, and nutrition, while sustaining a crew of four analog taikonauts for a full year^13^. Published recent plans from the CNSA further demonstrate that China has surpassed the US and its allies in both scale and preeminence of these emerging efforts and technologies, especially as compared to NASA’s current programs^4,10,14^ (Figs. 1 and 2).

This paper will review those plans with a perspective on current and future US and allies space capabilities gaps in human performance and habitation systems. Section “Background and History” provides historical and geopolitical context essential for understanding how decisions regarding space program funding and international partnerships have shaped current capabilities in BLiSS. Section “Cosmonauts, Astronauts, and Bioregenerative Systems” gives an international overview of the development of BLiSS systems and testbeds, traces the history of NASA’s development of advanced bioregenerative space habitation systems, and shows how these developments, along with associated policy decisions and research and program cuts, led to critical gaps in current NASA capabilities. Section “CNSA, BLiSS, and Modern Bioastronautics” is focused on China’s current leadership in BLiSS development. It analyzes how the CNSA synthesized the work that NASA discontinued, other international efforts, and domestic innovation to successfully develop the Beijing Lunar Palace, as well as how China’s substantial investments in a robust BLiSS initiative fit into its broader lunar and space exploration strategy. Section “Challenges for Deep Space Missions and Human Habitation” underscores the critical knowledge gaps regarding deep space radiation effects on biological systems, and the key role that BLiSS solutions play in addressing these challenges, while Section “Endurance Class Human Space Exploration and Habitation” articulates the fundamental requirements—and current gaps—for BLiSS in supporting future “endurance-class” deep space missions. The paper argues that these gaps pose a strategic risk to US leadership in human space exploration. In order for the US to maintain international space competitiveness in the emerging domain of lunar exploration in the 21st century, it is both necessary and urgent that these gaps be addressed. Section “Recommendations and Broader Impact: BLiSS Habitation Technology Development” concludes with specific recommendations for facilities and program investments that are crucial for the deployment of mature BLiSS technologies^15^ in the coming decade.

## Background and history

In October 1957, Sputnik catalyzed a massive and immediate response across the globe^16,17^. Humanity’s first venture to create an artificial satellite was at first viewed and depicted as a great advancement^18^, but was subsequently framed as a threat for political reasons. Although Sputnik “simply” orbited the Earth while transmitting a beeping radio signal in a common FM bandwidth, the Soviet Union achieved a significant breakthrough with its launch and technology demonstration, inspiring global excitement about the possibility of a new era of exploration. American families would watch Sputnik fly over their homes and cities and use their small transistor radios to listen to Sputnik’s signal^19^, all while knowing that the USSR had tested and detonated their first ballistic nuclear weapon just over a month prior.

Eventually, however, Lyndon B. Johnson^20^ and other political leaders publicly expressed deep concern about Sputnik’s launch, characterizing it as a crisis for US national security and connecting Sputnik to Soviet development of intercontinental ballistic missiles. This alarm ignited a political and cultural firestorm in the US, affecting many aspects of American life. In response, the government made massive investments in science and technology programs at the national level, leading to the creation of NASA, NIH, and NSF^21^. By the fall of 1958, students returning to American public schools after summer break were being evaluated from a new perspective emphasizing STEM performance. Space exploration, as well as the newly formed NASA space program, quickly became a national priority, with scientific, societal, economic, military, and political ramifications reaching into the present. In the years following Sputnik, this reorientation culminated in the collective psyche of the “space race” or “Moon race” between the US and the USSR, and ultimately in the first human Moon landing by Neil Armstrong and Buzz Aldrin on the Apollo 11 mission (Fig. 1), just 16 days past the 4th of July 1969. Despite the competitive drive leading up to this achievement, it is important to note that even the context of the Cold War did not stop American and Soviet scientists from seeking avenues for cooperation in space at every turn. For example, the 1962 Dryden–Blagonravov agreement established three cooperative projects between the two countries: weather data sharing and launching of meteorological satellites, magnetic field mapping of Earth, and experimental communication using the ECHO satellite and possible launching of future communications satellites^22^. Further, a US–Russian joint Moon landing was actually US bipartisan policy as early as 1958, and in 1962, 47 percent of Americans supported space cooperation with the Soviet Union^23^. NASA also began cooperative space initiatives with a range of other countries around the world, especially in Europe. Indeed, from the first meeting of the International Astronautical Congress in 1950, there was a strongly established transnational network of space scientists and engineers that regularly collaborated across borders, and this was a fundamental driver of scientific and technological progress.

In the ensuing decades, public and political support for large-scale missions of this class waned on both sides of the Iron Curtain. After the repeated N1 rocket failure in 1969, the Soviet Union gave up on completing a crewed lunar mission, instead focusing on their burgeoning and successful Salyut space station program. Based on the reconsolidation of the Soviet Almez military space station program, Salyut evolved into Mir. The American Apollo program (1961–1972) completed nine crewed missions, including six successful human lunar landings, but was phased out by President Nixon in the early 1970s to develop reusable spacecraft systems in the form of the Space Transportation System (STS), the official name for the space shuttle. During the STS era, NASA’s efforts focused on microgravity and spaceflight in low-earth orbit (LEO). This eventually led to the International Space Station (ISS) program, in which the former competitors in the lunar space race—the US and the USSR—now cooperated with each other as well as Europe, Japan, and others to build and operate the ISS. Later, after the retirement of the Space Shuttle in 2011, American and international ISS crews would all launch to the ISS using the former Soviet Soyuz spacecraft, operated by the “new” Russian space agency Roscosmos. During this period of intense, open, and unambiguous cooperation among NASA, Roscosmos, and others, the US space program benefited greatly, absorbing many of the lessons learnt from the early Salyut space station program, starting with Shuttle/Mir and then transitioning into the ISS era. This changed with Russia’s annexation of Crimea in 2014, which marked the beginning of the unraveling of the US/Russian ISS collaboration, and currently, the Russian and American programs have no plans for future cooperation beyond their outstanding obligations to the ISS program.

Despite the decline in the US-Russian space relationship, in the current era of accelerating space activity, there are hundreds of state and commercial-led space projects and missions rooted in international cooperation. These include two major state-led international space lunar initiatives, both focused on re-establishing a human presence on the Moon, on its scientific exploration, and on lunar resource extraction and utilization. The first is the Artemis Accords^24^ (AA), led jointly by NASA and the US State Department. Launched in October 2021 with eight original signatories, the number of participating countries in June 2025 stood at 55^25^. Based on the 1967 Outer Space Treaty^26^ (OST), the AA seeks to extend the OST through establishing shared principles for space exploration and resource utilization, particularly for lunar activities, while also stressing international cooperation and recognizing and encouraging private sector participation. While NASA’s Artemis program is separate from the AA and involves a separate bilateral agreement with NASA, signing the AA is required for participation in NASA’s Artemis program, which has a number of international partners, including JAXA and ESA. The second is the International Lunar Research Station (ILRS), led by China and Russia, which, in March 2021, signed a Memorandum of Understanding^27^, possibly in response to the AA, reflecting their growing space partnership over the last decade. The “ILRS Guide for Partnership”^28^, outlining the ILRS’s different phases and scientific objectives, followed in June 2021. In 2024, Russia’s cooperation was signed into Russian law^29^, just a month after Russia announced its nuclear energy plans for powering the ILRS, also in collaboration with China, and in September 2024, reports emerged indicating that India, a signatory of the AA and historically also a rival nation to China, is considering joining this effort^30^. The ILRS agreement currently numbers 13 signatories in total, with strong efforts by China to recruit additional partners, especially from the Community of Latin American and Caribbean States (CELAC). China is also devoting efforts to establishing collaborative space initiatives, such as the April 2024 China-CELAC Space Cooperation Forum^31^.

The recent emergence of these alliances raises concerns that these strategic partnerships could have potential long-term consequences for space governance and result in an increasingly complex and divided space geopolitical landscape, especially against the backdrop of the 2022 US sanctions against Russia, due to the Ukraine war, and the 2011 Wolf Amendment^32^, banning NASA/CNSA cooperation without congressional approval. The Sino-Russian ILRS collaboration is in a position to leverage the strengths of both nations, Russia’s legacy as a major space power, coupled with its long experience and expertise in space travel and international cooperation, and China’s increased monetary support for its space program. China’s space budget for 2024 was estimated at 19.89 billion US dollars^33^, an increase of 40.57% over the previous year. However, the Chinese space program is integrated with its national defense sector, with this potentially adding to its total space budget, and the actual numbers, therefore, remain somewhat opaque. China’s official national budget is surpassed only by the US, with total US government space program expenditure for 2024 estimated at 79.68 billion, an increase of 8.85% over the previous year. 24.875 billion of this constitute NASA’s 2024 budget^34^, and the currently proposed cuts would decrease this number by 24.19% to 18.8 billion for 2026^35^, leaving NASA with its smallest budget (adjusted for inflation) since 1962^36^. However, the fact that the 2024 budget represents only 0.4% of the US’s total federal budget, as opposed to the Apollo era’s 4%, has raised some concerns about NASA’s fiscal capability to execute its strategic plans^37^, adding to already existing worries about the US’s ability to stick to the Artemis schedule. In February 2024, China released its latest annual Blue Book^38^, outlining its ambitious space plans, including putting taikonauts on the Moon by 2030. This is around the same time at which the ISS will be decommissioned, leaving China, thanks to its Tiangong space station (TSS), poised to potentially become the only nation maintaining a continuous human presence in Earth’s orbit (although several commercial orbital space station endeavors are currently underway). The Artemis program has its own ambitious plans in the form of the Lunar Gateway Station, a small multi-purpose outpost in near-rectilinear halo orbit around the Moon, to allow access to various lunar locations. However, as Gateway is designed to serve as a stopping point for potential lunar and other deep-space missions, it is intended only for short-term crew visits of 30–90 days and would be uncrewed the majority of the time^39^. Regardless, the proposed 2026 budget includes Gateway’s cancellation, and so its future is currently uncertain.

While the possibility of the consolidation of two alliance blocs raises concerns, it is important to remember that, contrary to militaristic views of “space as the next battlefield,” the international space community remains laser-focused on cooperation^40^. At every annual meeting of the International Astronautical Congress—the largest gathering of space practitioners in the world—the main, high-level message is that international cooperation plays an indispensable role not only in maintaining space as a peaceful domain for all of humankind, but also for scientific advancement itself. As the paper analyzes challenges for US space leadership and NASA capability concerns for deep space missions, bioregeneration, and habitation systems, it will come back to the importance of international cooperation in addressing these critical needs.

## Cosmonauts, astronauts, and bioregenerative systems

The idea for building human life support ecosystems for space based on bioregenerative principles has been described and embraced throughout the emergence of human spaceflight and rocketry. Early on in the evolution of Russian aerospace and astronautics efforts, Konstantin Tsiolkovsky (1857–1935) wrote about agricultural approaches to life support for space exploration and habitation^41,42^. His work in aerospace design has been credited with inspiring the Soviet space program, and he developed both the engineering principles and the conceptual framework for advanced rocketry, human space exploration, habitation, and settlement. His numerous works contain everything from rocket thrusters, multistage launch rockets, and space stations to closed-loop bioregenerative systems for human life support, with his conceptual work ultimately envisioning lunar and Martian habitation as stepping stones for a sustainable human presence in space. It was understood from the beginning that the logistics for sustaining and (re)supplying humans in remote locations would pose a significant challenge and that transportation of supplies and resources for life support was going to be prohibitive. In response, the Soviet science program initiated several large-scale bioregenerative support programs, followed by programs in the United States, Japan, and Europe (for a review, see Wheeler 2017^43^).

Notable testbeds for bioregenerative research in approximate chronological order include the Russian BIOS-3 facilities (1960s to present), NASA’s Biomass Production Chamber, NASA’s Lunar Mars Life Support Test Project, Japan’s Controlled Ecological Experiment Facility, the ESA’s MELISSA Pilot Plant, the Canadian controlled environment research facilities at the University of Guelph, the Chinese Lunar Palace 1 facility in Beijing, and most recently the German Space Agency’s EDEN ISS project in Antarctica^43^. The Russian BIOS-3 program was foundational for the field, conducting some of the earliest studies of bioregenerative systems. It ran for nearly 30 years with a core test/demonstration facility and included multiple successful human subject experiments. While NASA could make a strong claim that it supported more bioregenerative research over the years, it never achieved an integrated test system like BIOS-3. The European MELiSSA program was initiated in 1989, making it the longest continuously running BLiSS research program in the world, with a considerable body of scientific output. Another notable effort was the privately funded Biosphere 2 project near Tucson, AZ. Part of Biosphere 2’s mission was to explore possible space applications for the future, although most space agencies considered the scale and mass of the system too large for near-term applications^43^. Nonetheless, Biosphere 2 provided valuable findings that will be applicable for future bioregenerative life support^44^.

While NASA already reviewed the need for bioregenerative systems in the mid-1960^45,46^, it did not begin extensive research until it formed the CELSS Program in the 1980s^47^, intended as the foundation for NASA’s Advanced Life Support (ALS) program^48^ (1989–2004). NASA’s CELSS Program sponsored university research grants and carried out directed research and development at Ames Research Center, Kennedy Space Center, and Johnson Space Center^49^. The intent was to develop low technology readiness level (TRL) research in university settings and then scale up in order to conduct “relevant” environment testing at Kennedy Space Center’s Breadboard Project^50^, followed by full integration testing with humans at Johnson Space Center^51^. TRLs for these whole systems tests were not formally tracked, but the intent for moving through fundamental research to scale tests and finally ground-based human integration tests was there. Some specific flight experiments were conducted with plants such as wheat, potatoes, and leafy greens^52,53^ and the concept for developing a TRL-like scale for candidate space crops was developed^54^.

At the time, NASA’s CELSS program was at the forefront of sustainable agricultural systems for space exploration, with several of its innovations foundational for the development of CEA^55^. For example, the concept of using light emitting diodes (LEDs) was patented through NASA research at the University of Wisconsin in 1990^56^. Soon, NASA’s Kennedy Space Center and companies like the Mitsubishi Corporation also began testing LEDs with plants^57^, resulting in significant advancements in the development of LED lighting systems for plant growth^58–60^. While hydroponics had been used for plant cultivation at least since the 1930s, and large-scale use of hydroponics for crop production in greenhouses expanded around the world in the 1970s^61^, NASA tailored hydroponics^62,63^ for crops such as potato, sweet potato, and wheat. The combination of using volume-efficient stacking of hydroponic trays and electric light banks likely arose from concepts developed at the University of Connecticut^64^ and was, beginning in 1987, used in NASA’s multi-tiered Biomass Production Chamber, arguably the first sustained vertical farming in the world^65^. Note that large-scale plant factories, such as Phytofarm in DeKalb, IL had been operated in the US and likely Japan prior to this^66^, but were not the multi-tiered systems used in modern day vertical farms and, thus, one may regard NASA’s efforts as facilitating the first use of vertical farming techniques for volume-efficient crop production^43^. These technologies underwent testing in large-scale testbeds, including at the Biomass Production Chamber at Kennedy Space Center^48^.

The CELSS program led to the NASA BIO-Plex^67^, an integrated bioastronautics program, canceled because of budget limitations from SLS and Orion as part of ESAS in 2004. Functionally, the BIO-Plex was developed as a fundamental food production, life support, and habitation system. Part of a test facility constructed at Johnson Space Center in Houston, TX, until funding was terminated and the facilities decommissioned due to federal building regulations, the BIO-Plex was one of the first-of-its-kind attempts to answer fundamental scientific habitation questions involved in developing a planetary or lunar surface base^67^. The BIO-plex’s food production systems would have been responsible for supplying plant foods, a breathable atmosphere, and potable water to the crew, tailored to the chosen mission scenario. Long duration space missions would have required development of both transit and surface exploration versions, with the two systems intrinsically tuned for different gravity conditions: the transit vehicle intended to operate in microgravity with mostly stored foods, while surface exploration operations would be used in partial gravity (hypogravity), producing food, oxygen, and potable water from crops grown in the facility. The transit system would rely upon prepackaged food with extended shelf life, supplemented with salad crops that would be consumed fresh^68^. Since microgravity imposes significant limitations on the ability to water plants and handle food, and allows only for minimal processing, the surface exploration facilities would grow and process crops such as wheat, soybean, rice, potato, peanut, and salad, to provide a nutritious and acceptable diet for the crew^69^. In addition to the constraints imposed on food production from the crops (e.g., crop variation, availability, storage, and shelf-life), there are also significant requirements for crew meals (e.g., recommended dietary allowances, high quality, safety, variety). For example, the LEO radiation environment of the ISS shortens the shelf-life of stored vitamin C supplements to less than one year^70^—even shorter in deep space—so vitamin C will have to be supplemented by plants. As food/nutrition becomes a fulcrum in human performance during spaceflight, food and nutrient production is a significant technology gap. The challenge is to create the right connections between crops and crew meals while dealing with issues of integration within a closed system, such as bio-safety, waste processing, water usage, and bioprocess engineering^71^.

## CNSA, BLiSS, and modern bioastronautics

As the US space program shifted its priorities towards the SLS/Orion transportation system, the CELSS/BIO-Plex program was phased out, with no current plans for rehabilitation. In the meantime, the CNSA, over the last 20 years, has embraced and advanced the very bioregenerative life support programs originally developed and then abandoned by the US^67^ (Fig. 2). NASA’s CELSS program delivered mature and executable plans for a large-scale, fully integrated BLiSS testbed in the form of BIO-Plex. Drawing on these publicly available plans, other international research, and its own technological development efforts, the CNSA has made substantial investments in BLiSS^4^. It adapted NASA’s fundamental work on core technologies for closed-loop ecosystems^72–74^ and, synthesizing international research foundations with domestic innovation, created BLiSS technology that eventually culminated in the successful ground-based development of their lunar habitation system known as the “Beijing Lunar Palace”^13^. The Beijing Lunar Palace program thus translated the foundations and outputs of the discontinued NASA CELSS program into a viable habitation technology for a lunar outpost. Unifying existing efforts and its own scientific and technological advances, the CNSA has successfully demonstrated closed-system operations for a breathable atmosphere, water, and nutritious food for a crew of four taikonauts for an entire year^13^, thereby gaining critical user experience for actual deployment in space. Yet, even this groundbreaking effort failed to close the loop on waste recycling.

These successful proof-of-concept studies, completed in 2016, have paved the way for further expansions of CNSA’s bioregenerative life support programs and now serve as the foundation for China’s coming lunar outpost. Published plans aim for beginning construction of the ILRS in the 2030s, following a series of demonstration missions before the end of this decade^75,76^, including two missions to the Moon’s south pole around 2026 and 2028, focusing on demonstrating 3D bricks for habitat construction printed from lunar regolith^76^. The CNSA programs in fundamental BLiSS biotechnology development are scientifically robust, programmatically funded as key strategic capabilities for advancing the ILRS, and benefit from access to several decades of BLiSS research championed and supported by Russia. The CNSA has adopted a strategic, phased approach to steadily develop its space capabilities and establish a permanent presence in low Earth orbit. While there is no detailed budget information about its different programs, there is significant research and development, with a special emphasis on BLiSS technologies^77^, coming from CNSA programs in “taikonautics systems”. For example, the TSS now boasts advanced BLiSS research capabilities comparable to those based on the ground, including crop production and plant research capabilities, as well as fundamental biotechnology and biomedical research facilities. The CNSA programs in plant space biology have also benefited greatly from collaboration with the German Space Agency (DLR)^78^ in conducting several spaceflight experiments on CNSA launch vehicles^79^. Other key firsts for the CNSA space biology program include the first mammalian embryo development experiments in space^80^ and the first biological payload on the lunar surface with the first seed germination and plant growth experiments on the Moon^81,82^.

Thus, the Chinese are now benefiting greatly from their focused and successful space station program. The program’s first phase began with the Target Vehicles phase, consisting of the Tiangong-1 prototype module launched in 2011, for target, rendezvous and docking practice, which hosted two crewed missions, Shenzhou-9 and Shenzhou-10 (deorbited in 2018). Phase 2’s Tiangong-2 (2016–2019) was a space laboratory module for the Shenzhou-11 crewed mission, which tested life support systems and conducted scientific experiments before being deorbited. The current and final phase (2021–present) is the multi-module TSS, with the aim of establishing a permanent, crewed presence in low Earth orbit. This phase began in 2021 with the launch of the Tianhe core module—the central living and working space for taikonauts—followed by the Wentian and Mengtian laboratory modules in 2022, significantly expanding the station’s research capabilities. New information about the TSS and its advanced research systems and capabilities is emerging constantly. The CNSA’s Long-March launch systems exceeded performance expectations, with backup modules now available for the CNSA to execute expansion and implement long-term operations. China’s June 2025 zero-altitude test of its next-generation modular crew spacecraft, Mengzhou, validated its launch‑escape system by rapidly propelling it away from the pad, safely separating the crew capsule and completing a secure landing. This achievement marks a further milestone in China’s lunar ambitions, demonstrating critical crew safety capabilities for both Low Earth Orbit and planned lunar missions. Tiangong’s future involves the addition of new modules, including plans for a larger core module and specialized modules for bioregenerative systems production and scientific research^83,84^. The overarching goal of these endeavors is both to maintain a continuous human presence in space, furthering China’s foothold in long-duration missions, but also to conduct cutting-edge BLiSS research^84^, crucial to supporting any such extended presence.

In stark contrast to the thriving Chinese program^85^, these key advances and demonstrated BLiSS capabilities have not been addressed by the American program, which does not currently include plans to rebuild the NASA programs canceled in 2004^15^ (Figs. 1 and 2). The US space program, therefore, faces a multi-year challenge merely to revive and rebuild the required facilities and infrastructure. Moreover, the ISS has been severely underutilized by NASA for advancing bioastronautics science and engineering capabilities for bioregenerative life support. With half of ISS utilization redirected to the ISS National Lab for the commercialization of LEO, NASA’s plans for leaving LEO and developing bioastronautics capabilities to return and eventually maintain a crewed habitat on the Moon were further curtailed. The US national program thus lacks the key bioastronautical facilities and capabilities for human habitation systems that integrate CEA and bioregenerative manufacturing of fundamental life support elements. Now on the verge of returning to the Moon, NASA needs to develop the critical capabilities required to build and operate a lunar outpost. Meanwhile, the recent collaboration between the Chinese and Russian space programs presents a formidable partnership. Just as the US benefited from the last twenty years of joint programs between NASA and Roscosmos, the recent collaboration between the Chinese and Russian space programs will benefit from decades of Russian space experience as well as the rapid—and well-funded—advancements of the Chinese program.

## Challenges for deep space missions and human habitation

**Fig. 3:**
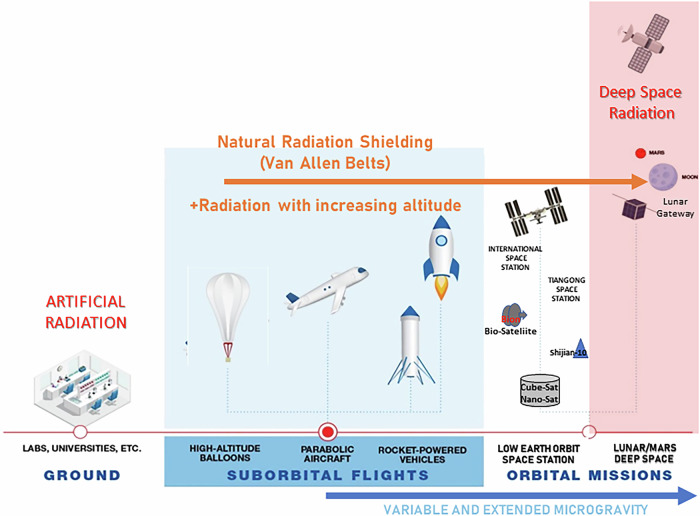
Radiation research conditions and platforms that are encountered and used in space missions and research. Artificial radiation sources lack the qualitative and quantitative characteristics of naturally variable sources. Radiation is a continuum of exposure transitioning from the surface of the planet where we are shielded from major aspects of galactic cosmic radiation in deep space. We continue to learn about the biophysical challenges of deep space exploration, a major focus of research for human medical countermeasures in LEO, but now more important as a mission-critical component in upcoming lunar missions that will extend out beyond the Van Allen belts (modified from original source^112^).

As future missions aim to go beyond the radiation shielding of LEO, the next generation of 21st century space explorers will require BLiSS technologies for dealing with the threat of deep space radiation (Fig. 3) and because of the exponentially challenging logistics associated with such missions. These BLiSS technologies represent a fundamental new level of physical assets and bioastronautics capabilities required for deep space human habitation. The personal health sacrifice and commitment of the next lunar exploration crews, dealing with the biophysical challenges of deep space radiation, should not be underestimated. Over the last 20+ years, continuous spaceflight operations on the ISS in LEO have afforded the opportunity to conduct biological and biomedical research as part of NASA’s Space Biology, Biological and Physical Sciences, and Human Research Programs to develop countermeasures to protect crew in spaceflight operations. Radiation and microgravity both inflict a biological and medical cost on crew health, which in turn impacts human performance and mission viability^86^. As humans embark on lunar and deep space missions, they have to absorb and cope with galactic cosmic radiation^86^, and the “easy and slow” days of LEO will be gone. As of June 2025, only 24 individuals in the history of human spaceflight have left the protective shield of the Earth’s magnetosphere as part of the Apollo missions, facing radiation challenges beyond the relative safety of the low Earth orbit environment. Polaris Dawn^87^, in September 2024, briefly reached the first van Allen belt (although remaining inside LEO), thereby exposing four individuals to the highest radiation levels since the last Apollo mission, Apollo 17, in 1972, but still well below those that would be encountered in deep space. Extremely limited available human health data—especially outside LEO—means that we still know only very little about the true effects of deep space radiation. As a result, the experiences and data gathered from the Apollo missions continue to inform plans for future human missions to the Moon, Mars, and beyond. One recent study suggests that the Apollo lunar astronauts might be prone to significantly increased cardiovascular disease mortality rates^88^. Radiation effects remain a major source of uncertainty, and radiation protection, including protection through BLiSS measures, is therefore an area of critical concern.

Thus, even over fifty years later, and after almost twenty-five years of research on the ISS, the radiation environment beyond LEO still presents an existential biophysical challenge for long-duration space missions, not just for humans but also for other biological systems. Current spacecraft designs and radiation countermeasures are insufficient to protect the crew or critical biological systems from the harsh cosmic radiation in deep space. For successful BLiSS, any habitat structure must provide substantial physical protection to shield plants and other biological components from radiation exposure. Understanding how plants perform under the Moon’s radiation environment is crucial, as these findings could offer key insights into human health, particularly with respect to stress responses related to nutritional antioxidants such as vitamin E and vitamin C. Research on plant responses to cosmic radiation not only informs the design of future space habitats but also sheds light on broader biological mechanisms that may enhance human resilience in space. Investigating the performance of plants under lunar radiation conditions is, therefore, fast becoming a major research goal. NASA’s Lunar Effects on Agricultural Flora payload^89^, currently being developed as part of the Artemis 3 mission, is a first step in this direction, helping to refine BLiSS technology and to ensure its effectiveness in deep space.

Further, given the physical distances associated with lunar and deep space missions, ecologically engineered approaches for sustainable life support systems will be an existential necessity. The vast distances between the Earth and Moon create significant logistics issues for crewed operations, related to atmospheric regeneration and re-supply of food and water. Bioregenerative ecologically engineered systems are our best strategy to recycle and regeneratively resupply^71^ a working research outpost; without such systems, missions will be limited in duration due to the enormous cost of resupply launch services. The construction and operational maintenance of lunar or Martian surface habitats will be of highest consideration given the need to dedicate crew time to exploration and research outside of habitation systems.

## “Endurance class” human space exploration and habitation

The imminent era of deep space “endurance class” exploration requires new approaches and technologies to advance the science of human space habitation. Deep space habitation using bioregenerative approaches will enable operational capabilities in spaceflight systems that would be unattainable based on current ISS logistics and resupply models. The operational envelope of this new endurance class approach is significantly enhanced and enlarged when compared to notional concepts of operations from past lunar missions. Establishing an operational outpost on the lunar surface would enable crewed operations at the very frontiers of human exploration. This, in turn, would provide an important test and validation for concepts that could then be deployed for Mars missions^90^. By utilizing bioregenerative systems and by ecologically recycling the life support systems of the habitat itself, operational timeframes and logistical footprints are significantly enhanced. The next generation of space explorers will need to be able to rely upon bioastronautics approaches that integrate CEA, space bioprocess engineering, and personalized medicine, including through the application of integrated omics technology pioneered by NASA GeneLab^91–93^.

Since such bioregenerative systems are a clear necessity for a sustainable human presence in space, its associated critical technology gaps directly limit future lunar and Martian missions. This was understood clearly during the early days of space exploration, which is why all space programs—Roscosmos, NASA, ESA, JAXA, CNSA, and others—have supported and are supporting varying degrees of BLiSS component technology. Yet, despite the recognized importance of these capabilities, there are currently no official programs, besides the CNSA’s, geared towards the development of a fully integrated, closed-loop bioregenerative habitat. While there have been partial systems tests, over limited periods, to date, no nation has demonstrated a completely closed BLiSS system that integrates or even includes the essential requirements for regenerative operation, feedstock and nutrient recycling, and human waste processing.

Recognizing the urgency of addressing these gaps, the development of BLiSS technologies through a dedicated research campaign, funded through an increase in NASA’S Biological and Physical Sciences budget, was also one of the two major recommendations of the 2023 US National Academies of Sciences, Engineering, and Medicine (NASEM) Decadal Review^15^ for the future of biological and physical sciences in space. This report emphasized the need for robust BLiSS as a strategic priority for NASA, as well as an increase in research in fundamental biophysics, another area critical to BLiSS reliability in space and spaceflight operations. Emphasis on BliSS technologies is also in alignment with NASA’s recent *LEO Microgravity Strategy*^94^, its *Moon-to-Mars* Architecture Definition Document^95^, and its overall strategic plan, especially *Goal 2*, focused on an extended human presence on the Moon^96^. Requirements for future lunar missions will require biosustainable life support systems as fundamental capabilities for supporting human health and well-being during long-duration missions beyond low Earth orbit. By harnessing biological processes and resources, bioregenerative systems can provide essential needs such as food, water, and oxygen while effectively recycling waste, thus minimizing resource input and enhancing mission sustainability^71^.

## Recommendations and broader impact: BLiSS habitation technology development

Based on urgent and critical needs, and in line with the above-mentioned reports, strategic plans, and programs, we therefore urge the following recommendations.

### Recommendation 1: protoflight habitat

NASA and other space agencies/interested groups need to develop accurate test facilities for bioregenerative systems that integratively build upon a base physico-chemical human habitation with existing ECLSS capabilities, to understand a sequential transition from such systems to BLiSS habitation on future missions^97^. In addition, physico-chemical ECLSS will serve as a “solid-state” back-up and buffer for controlling and adjusting system performance in lag or transition phases of bioproduction and growth. These ground test facilities need to be operated for several years to understand reliability and failure issues, and to get a better understanding of sustainability. In particular, a prototype lunar outpost for human-rated systems needs to be constructed and operated, with extensive research and development efforts required to validate the technology. According to some estimates, 4–8 years of operational experience is necessary to achieve a full TRL status for a fully bioregenerative habitat. Key recommendations for the development of a bioastronautics habitat include adopting a full protoflight development approach using a terrestrial analog facility as a testbed to integrate genomic biotechnology (omics) for CEA, microbial ecosystems, and waste recycling as part of a closed human habitation system, as well as personalized medicine. Comprehensive waste recycling represents the most significant technical challenge in bioregenerative systems and has been inadequately addressed in existing research efforts, paradoxically neglecting an area where space technology development could directly address one of Earth’s most pressing sustainability challenges. Overall, the focus should be on full-fidelity mission architecture and operation testing at the endurance-class deep-space habitation systems level. The protoflight habitat should be functionally operational for a full year before the commencement of the lunar outpost.

### Recommendation 2: biophysical BLiSS research and platforms

To engineer spaceflight systems that perform effectively in spaceflight conditions, it is necessary to understand the impact of spaceflight on the biological elements of a BLiSS system, which necessitates ground and microgravity platforms for BLiSS research^98^. Research emphasis on fundamental exploration-enabling technology will require new basic and applied research to differentiate between radiation and microgravity effects and their corresponding, respective countermeasures. This includes research into understanding how altered gravity environments affect physical phenomena, such as fluid dynamics^99^, which in turn has consequences for biophysical mass transport and other biological processes^100^. Addressing deep space radiation and chronic exposure to reduced gravity in both deep space transit as well as surface habitation will not only necessitate significant research directed toward fundamental BLiSS technologies, but also towards reestablishing full scientific utilization of the ISS or future commercial space stations. Specifically, we recommend well-instrumented scientific control experiments to differentiate between microgravity and radiation effects through the implementation of 1g and partial gravity in-flight controls based on constant centrifugal acceleration, designed to simulate lunar and Martian conditions.

### Recommendation 3: BLiSS omics systems

The scientific objectives of the BLiSS program necessitate the use of modern omics technologies to facilitate fundamental and applied systems, ultimately leading to the development of feedback and control technologies for habitat performance monitoring and regulation. The establishment of a robust translational program through NASA GeneLab, a data and analysis portal for spaceflight and ground control omics data, will enable ground-based translation of previous spaceflight experiments, fostering new research ideas to advance technologies arising from these novel opportunities. An integrated BLiSS/omics platform is envisioned as a critical component of modern bioastronautics, which fuses precision agriculture, microbiome/bioprocess systems engineering, and personalized medicine. The emergence of BLiSS/omics-based bioastronautics directly addresses previous criticisms of bioregenerative systems as “black boxes” that cannot be integrated into reliably controlled engineered systems^101^.

### Recommendation 4: convergent funding and international cooperation

The interdisciplinary nature and broad societal benefits of bioregenerative human habitation technology necessitate and justify the involvement of a diverse community of stakeholders in funding these programs, including international partners. This is an area ripe for international cooperation. Prior to ESAS, NASA Code *U* was funded at just over $1B/year^15^ to support the program portfolio for ground- and space-based investigations, as well as to advance the CELSS program, up to the canceled trials of BIO-Plex in 2004. Given the demonstrated spinoff value of NASA’s CELSS in creating the current CEA industry, it is imperative to consider funding sources beyond NASA. BLiSS system technologies are directly applicable to the NIH, USDA, EPA, DoD, and Department of Commerce, in addition to NASA, DARPA, USAF, and USSF^102^. Convergent funding will facilitate immediate spinoff output, advancing fundamental human health, agriculture, and sustainable environmental ecosystems.

International partners, such as the signatories of the AA, should also consider contributing to these efforts, not only through funding, but also through multinational scientific cooperation. For example, the Canadian Space Agency (CSA) and ESA are already contributing major components for agricultural bioproduction for Artemis. More generally, history has shown that international cooperation in the space domain has been a powerful driver of scientific and technological advancement, not just with its poster child ISS but its many other large-scale collaborative projects, such as the joint NASA/ESA/ASI (Agenzia Spaziale Italiana) Cassini-Huygens mission or the NASA/CSA/ESA collaboration on the James Webb Space Telescope, to cite just two. The same is true of BLiSS technologies^43^. NASA, as the world’s leading space agency, should play the leading role in these developments, but international collaborations will bolster the scientific pace significantly. The growing number of space agencies—nearly 80 as of June 2025—presents unprecedented opportunities for collaborative research and for ensuring the safe and sustainable presence of humans in space, regardless of their origin or destination. Further, such cooperative efforts can also amplify the Earth-side benefits of space-developed technologies, especially in areas like sustainable agriculture and closed-loop resource management, both of which are of growing global importance and essential to humanity’s future on our home planet.

### Broader impacts and relevance

The lack of availability of BLiSS technologies and systems—both at the governmental and commercial levels—currently limits the objectives of human-crewed lunar exploration programs. BLiSS technologies represent a critical technology gap that must be addressed in order to enable a robust lunar exploration and development program. This will facilitate the establishment of a human-tended outpost with bioregenerative capabilities for a crew of four, serving as an expeditionary base that would enable humans to create a permanently crewed outpost while simultaneously fostering a new biotechnology sector that enables bio-based manufacturing. It further supports the US’s *Space Priorities Framework*^103^ and a number of US federal initiatives geared towards developing a vibrant cislunar economy, such as the White House Office of Science and Technology’s *National Cislunar Science and Technology (S&T) Strategy*^104^, NASA’s *Artemis*^105^ and *Moon-to-Mars*^95^ *programs*, and DARPA’s *10-Year Lunar Architecture (LunA-10) capability study*^106^. This approach requires that NASA and NASA’s partners develop a roadmap to evolve from early exploration low-cost ‘camping trips’ to sustainable surface habitats able to efficiently reuse and recycle resources^107^. The promotion of BLiSS biotechnology and biophysics also advances other critical areas of microgravity sciences, particularly those related to mass transport and diffusion in space, which currently constrain zero-boiloff cryogenics, a critical technology for spacecraft fueling in space. Advancing basic science research can generate innovations that can be applied broadly across disciplines and systems.

Further, the “spin-off” benefits from the development of a lunar base will be realized on Earth well before the execution of any actual deep-space habitation mission. The advancement of the required novel technologies for CEA, human-built environments, and precision medicine can achieve nearly immediate application and adoption by the robust and extensive agricultural, environmental, and medical industries on Earth. The development of BLiSS technologies—whether ultimately geared towards deep space human exploration or terrestrial sustainability—is a major contributor to solving global ecosystem challenges, with bioregenerative design and engineering crucial elements in advancing social and environmental resilience. Sustainable development is increasingly critical in addressing global challenges, as highlighted by the United Nations Sustainable Development Goals (SDGs)^108^. These 17 interconnected goals, also adopted by US-AID^109^, aim to tackle pressing issues such as poverty, inequality, climate change, environmental degradation, peace, and justice. As humanity embarks on ambitious deep space exploration missions, aligning these efforts with the SDGs, as well as ethical considerations more generally^110,111^, is essential to ensuring that advancements contribute positively to humanity.

The US NASEM Decadal Review^15^ underscores the integration of bioregenerative research into NASA’s exploration framework, advocating for innovative technologies that align with both the UN SDGs and the goals of deep space exploration. By investing in bioregenerative life support research, especially in NASA’s *Moon-to-Mars* program^95^, NASA has the opportunity to lead in developing systems that not only support astronauts in lunar and Martian environments but also contribute to sustainable practices on Earth. This dual focus on space exploration and terrestrial sustainability represents an important step toward ensuring that our quest for knowledge and discovery in space aligns with our responsibility to protect and enhance the quality of life on our home planet. In the past, NASA has led the way with its Apollo, shuttle, and ISS programs, leveraging science and technology for the benefit of humankind. It now has the opportunity to secure American leadership in the future of human space exploration through a cooperative endeavor to build a permanent human outpost on the Moon—the next big leap. The benefits of new science and knowledge are unknowable until they are discovered, and living on the Moon is the next great challenge to catalyze humanity to innovate and create at the frontiers of existence, space.

## Data Availability

No datasets were generated or analysed during the current study.
